# Illness perception and cardiovascular risk factors in patients with myocardial infarction undergoing percutaneous coronary intervention in Iran

**DOI:** 10.1186/s12872-022-02684-9

**Published:** 2022-06-02

**Authors:** Aysan Thagizadeh, Akram Ghahramanian, Vahid Zamanzadeh, Naser Aslanabadi, Tonia C. Onyeka, Nargess Ramazanzadeh

**Affiliations:** 1 Students’ Research Committee, Tabriz University of Medical Sciences, Tabriz, Iran; 2 Department of Medical-Surgical Nursing, Medical Education Research Center, Health Management and Safety Promotion Research Institute, Faculty of Nursing and Midwifery, Tabriz University of Medical Sciences, Tabriz, Iran; 3 Department of Medical-Surgical Nursing, Faculty of Nursing and Midwifery, Tabriz University of Medical Sciences, Tabriz, Iran; 4 Cardiovascular Research Center, Tabriz University of Medical Science, Tabriz, Iran; 5 Department of Anesthesia/Pain and Palliative Care Unit, College of Medicine, University of Nigeria, Ituku-Ozalla Campus, Enugu, Nigeria

**Keywords:** Cardiovascular disease, Illness perception, Risk factor, Myocardial infarction, Patient education

## Abstract

**Background:**

Knowing of perception of the illness, and cardiovascular risk factors in patients with myocardial infarction is crucial in engaging in effective secondary prevention. This study aimed to examine illness perception and cardiovascular risk factors in patients with myocardial infarction undergoing percutaneous coronary intervention.

**Methods:**

The participants comprised 131 patients undergoing a first-time percutaneous coronary intervention at a metropolitan, tertiary referral hospital in Tabriz, Iran. The convenience sampling method was employed to select the research sample within a six-month period. The instruments used were as follows: (1) Demographic and health information form, (2) The Brief Illness Perception Questionnaire (3) The Health Risk Assessment framework developed by the Centers for Disease Control and Prevention. The design of the study was descriptive, cross sectional. The continuous variables were analyzed using Independent t-test and analysis of variance (ANOVA); and categorical variables were compared using the chi-square test.

**Results:**

Most participants had a positive family history of cardiovascular disease (54.2%), with 66.4% of participants having at least one cardiovascular risk factor such as diabetes (36.6%) hypertension (32.8%) and dyslipidemia (16%). Most participants were physically inactive (78.6%), about 48.9% were overweight, 34.4% suffered from obesity and 26% were smokers. Illness perception in this study was seen to be high (6.21), with highest scores occurring in the illness control dimension (6.83) and lowest scores occurring in the understanding dimension (3.77). There was a significant relationship between illness perception and physical activity, nutrition, sleep and general health. Direct significant relationships between biometric values (cholesterol, glucose, blood pressure); psychological factors (depression, anxiety and stress) and illness perception were also found to exist.

**Conclusions:**

Low scores in two dimensions of illness perception may lead to psychological consequences such as stress, anxiety, and depression. The relationship between illness perception and some risk factors of cardiovascular disease such as physical activity, diet and biometric values, reveal the need for more attention to patient education and counselling.

## Background

Characterized by an annual mortality rate of about 17.9 million people worldwide as at 2016 [1], cardiovascular diseases (CVDs) remain one of the commonest causes of death globally, accounting for 20% of worldwide deaths and 35% of deaths in Iran [2, 5]. Myocardial infraction (MI) has been known to cause more than four-fifths of deaths from cardiovascular diseases [3]. Compared to Western nations, most Asian countries have higher mortality rates of CVDs except for Japan, South Korea, Thailand, and Singapore [4]. Given the high costs of treatment for patients with MIs and the high burden of expenses on the healthcare system [5], an important but low-cost method of controlling CVDs that governments can adopt is to focus on mitigation of relevant risk factors, especially the modifiable factors [6, 7]. Several studies support the role of risk factor change on the quality of life of patients at high risk of cardiovascular events [8, 9], with several other studies revealing that people know little and do little about cardiovascular risk factors. These findings have spurred identification of modifiable CVDs risk factors in order to mitigate these medical conditions [10–13]. More than 70% of CVD cases globally are attributed to modifiable risk factors [14]. From the perspective of self-determination theory, positive human behaviors that are aligned with health-related goals are usually internalized by the patient in question, increasing motivation and making the patient feel more responsible for the outcomes, a situation that suggests more attention be given carefully to the patient’s experience and patient’s illness perception [27]. The importance of illness perception with respect to myocardial infarction has been demonstrated in the high attendance at rehabilitation by patients who feel they have a good grasp of the nature of the illness and that it can be controlled, a phenomenon related to patients’ compliance and relevant to patient-reported outcomes [28]. Thus, a patient who believes that nothing can change the course of the disease might be more prone to risk [29]. Patients can successfully adapt to CVDs only if they make long-term changes to their lifestyles and change their false beliefs regarding their conditions [21]. In addition, adherence of patients to secondary preventive measures may reduce the risk of progression of coronary heart disease (CHD) and speed up the recovery progress [1].

The concept of illness perception, a relevant factor in cardiovascular health behavior [1], is an organised belief model that predicts the patient’s future behavior toward the disease management and the correction of risk factors [15]. Based on several studies, a relationship has been established between illness perception and patients outcomes such as quality of life [16, 17], coping [18] and health care use [19]. This concept is derived from the Self-regulation Model of Leventhal [22] that states that in the face of an illness, an individual is moved to modify the health-related risks of that condition, according to how she/he perceives the illness. Also, there might even be different illness perceptions among patients with similar medical conditions and injuries [30]. Hence, a good perception or understanding of an illness leads to better adaptability, an improved general health status, ability to manage the illness more efficiently, a quicker return to work and reduced stress levels [20, 23, 24]. However, most patients with ischaemic heart diseases (IHDs) have insufficient information regarding the important role of this form of behavior plays in preventing the recurrence of IHDs, thus putting themselves at high risk of IHD recurrence [21]. Nicolai et al. indicate that changing lifestyle after an Acute Myocardial Infarction (AMI) is affected by a combination of physical, mental and social factors. They opine that such patients need more personalized information regarding causes of and risk factors for diseases as well as advantages of lifestyle change. They also advocate for involvement of friends and family members in the form of social support and participation during consultation and lifestyle training [22].

The opinions of patients about diseases often differ from those of therapists, with medical staff being largely unaware of patients’ opinions about their diseases and rarely focusing on the latter’s beliefs. Lack of information, inadaptability to various treatments, and skepticism towards the positive effect of behavior change on the outcome of a disease are correlated with negative self-care behaviours of patients [23]. Knowing patients’ risk factors and their perception of CVDs can provide clinicians and health managers with realistic insights for the development and implementation of public health policies to promote patient education regarding the reduction of adjustable risk factors and prevention of IHDs. In illness perception, patients’ beliefs regarding lifestyle and disease risk can be adjusted in clinical interventions, giving nurses and other clinicians the opportunity to develop purposive interventions that will correct such beliefs in patients [3], especially as changes in illness perception over time has been shown to be useful in aiding the development of educational programs to bring about positive attitudes towards health beliefs in this subset of patients [1]. Hence, this study aimed to determine the IHD risk factors and their correlations with illness perception among the patients hospitalized and diagnosed with AMI at Shahid Madani Cardiovascular Center (Tabriz, Iran) who received the percutaneous coronary intervention (PCI).

## Methods

### Study design and participants

The statistical population of this cross-sectional descriptive study included 131 patients diagnosed with AMI who visited Shahid Madani Hospital and underwent first-time PCI. This study covered all the departments of surgery and cardiology and Cardiac Care Units (CCUs) of Shahid Madani Hospital in Tabriz, Northwest of Iran. This hospital is a CVD sub-specialty center with facilities for cardiac catheterization and is a referral centre for patients in need of angiography or PCI. After obtaining permission from Tabriz University of Medical Sciences, patients who met the inclusion criteria, were enrolled into the study at the discretion of clinicians in the CCU and departments of surgery and cardiology. The purpose of the research was explained to the participants, who filled out the written consent forms individually, completed the questionnaires and were interviewed. The convenience sampling method was employed to select the research sample within a six-month period (from December 2020 to May 2021).

In order to estimate the sample size, the formula for the estimation of a mean was employed. In the current study, the value of z with a 95% confidence level was 1.96, and total mean (standard deviation) of IP was 45.45 (9.33) based on the study of Allahbakhshian et al. [24] and considering d (precision) equal to 0.55, a sample size of 119 people was obtained. Regarding a possible attrition rate of 10%, the sample size was equal to 130 people. Continued enrollment of participants was conducted until the planned sample size was reached.

### Inclusion criteria

- Adult patients aged 18–65 years.
- All patients experiencing their first myocardial infractions, receiving PCIs on an emergency basis for the first time at the hospital, who were in stable medical condition.
- Persian, Turkish, or Kurdish-speaking Iranians conversing in either Turkish or Persian languages.

### Exclusion criteria

- Exclusion criteria are: unwillingness to continue participating in the study, and failure to complete the data collection tools.

### Data collection

Data were collected by first author in order to minimise variability. The data were gathered via face-to-face interview using validated questionnaire. The *Health Risk Assessments (HRAs)* and *Brief Illness Perception Questionnaire (IPQ-B)* were used in this study.

### Health risk assessments (HRAs) questionnaire

The framework proposed by the Centers for Disease Control and Prevention (CDC) and called *Health Risk Assessments (HRAs)* was employed to evaluate the IHD risk factors. The CDC developed this framework on the basis of three recently conducted systematic literature reviews and expert input from physicians, researchers, members of medical associations, wellness program developers, and CDC subject matter experts [25]. This framework includes 15 dimensions (with 37 indices): physical activity, tobacco use, alcohol use, nutrition, seat belt use, depression, anxiety, high stress, social/emotional support, pain, general health, activities of daily living, instrumental activities of daily living, sleep, and biometric measures (*i.e.*, blood pressure, cholesterol, blood glucose, overweight/obesity). Among health risk assessment tools, the CDC’s HRAs framework may be completed through an interactive program before or as part of a visit and can identify chronic diseases, injury risks, modifiable risk factors, and urgent health needs. It assesses physical, psychological, and socioeconomic factors in addition to how these influence health and functional status [26].

All subjects were measured for their height, weight, body mass index (BMI), and blood pressure (BP) by first author. Height and weight were measured to the nearest 0.1 cm and 0.1 kg respectively using a pre-calibrated freestanding mounted to scales stadiometer (Seca, Germany) and height rod in light clothing and without shoes on. BMI (kg/m^2^) was calculated as weight (kg) divided by squared height (m2). Obesity is defined as BMI > 30 kg/m^2^ and overweight is defined as BMI of 25.1–30 kg/m^2^ [25]. An automated BP monitor (Citizen, Japan) was used to measure BP. Borderline high is defined as elevated BP with systolic BP (SBP) 120–139 mmHg and/or diastolic BP (DBP) 80–89 mmHg. High BP is defined as elevated BP with SBP ≥ 130 mmHg and/or DBP ≥ 90 mmHg [25]. Biochemical tests at the time of admission or the first day after hospitalization were requested for all patients by patient’s physician and their values were extracted from patients' electronic medical record. According to CDC’s HRAs framework, borderline high cholesterol is defined as elevated cholesterol 200–239 mg/dl and high cholesterol is defined as elevated cholesterol 240 mg/dl or higher. Borderline hyperglycemia is defined as elevated glucose 100–125 mg/dl and hyperglycemia as elevated glucose 126 mg/dl or higher. Also, according to this framework, those who have smoked in the last 30 days are defined as smokers [25].

The original version of HRAs questionnaire was translated from English into Persian. This was done by a faculty member with good command of the Persian language. The translated version was reviewed by the research team. Then, two faculty member familiar with measured concept and with good command of both languages reviewed the translated version and a final version was obtained. Intra-class correlation coefficient was between 0.78 and 1 for 15 dimensions of HRAs questionnaire in a pilot study, in which the questionnaire was completed for 10 patients by two members of the research team independently.

### Brief illness perception questionnaire (IPQ-B)

The IPQ-B is a nine-item scale that has each item rated on a scale from 0 (minimum) to 10 (maximum) and which assesses the emotional and cognitive aspects of individual’s illness [27]. Each of the terms of this questionnaire examines a dimension of understanding of the disease as follows: item 1: consequences, item 2: timeline, item 3: Personal control, item 4: treatment Control, item 5: identity, item 6: concern, item 7: illness comprehensibility and item 8: emotions, and item 9 is a question that is answered by the patient about one’s opinion regarding the cause of illness. Items 1–5 assess the cognitive dimensions which relate to understanding of illness, its causes, effect of treatment while items 6 to 8 evaluate the emotional dimensions that relate to emotions such as mood, fear, anxiety or anger. The total score of illness perception is calculated by inverting the score for items 3, 4 and 8 and added to the score of the other items. The maximum total score is 80 and the minimum total score is 0. A higher score indicates a more threatening view of the patient, while a lower score indicates a more optimistic view of the disease [28].

The reliability and validity of the Farsi version of this questionnaire has been verified and localized by Bazzazian and Besharat [29]. Their results showed that the scale has cross- cultural validity. In the present study, the internal consistency reliability (Cronbach’s alpha) of questionnaire’s subscales were between 0.76 and 0.82. According to the criteria (at least 0.65) indicated by Devellis [30], most subscales demonstrated good internal consistency reliability.

### Statistical data analysis

All data were analyzed using SPSS 21.0 (IBM, Armonk, NY, USA). The continuous data for normal distribution were tested using the Kolmogorov–Smirnov test, which showed the normal distribution of variables. The continuous variables are presented as means ± standard deviation and were analyzed using Independent *t*-test and analysis of variance (ANOVA) and the Least Significant Difference (LSD) post hoc test. Categorical variables are presented using n (%) and were compared using the chi-square test.

## Results

### Characteristics of patients

Of the 131 study participants, 72 patients (55%) were male, and 130 patients (99.2%) were married. They were aged 36–65 with a mean age (± standard deviation) of 55.06 (± 7.72) years old. The mean age of male patients was 53.25, whereas that of female patients was 57.28, and most of them were illiterate (57; 43.5%). A positive family history of CVDs was seen in 71 patients (54.2%), and 87 (66.4%) of them had at least one co-morbid medical condition, the most common being hypertension (HTN) (32.8%), diabetes mellitus (DM) (36.6%), and HPL (16%). The co-morbid entities of DM and HTN were jointly observed in 12.2% of participants, whereas 8.39% had all three comorbid conditions. All patients were presenting for PCI for the first time.

### Analysis of health risk assessment

Regarding IHD risk factors based on the HRA questionnaire, research results showed that 78.6% of patients reported no sports activities. The average weekly duration for engagement in sporting activity was 0.82 days, with the average time duration for their sports activities being 7.59 min. Moreover, 26% of them reported smoking in the past 30 days, and 24.43% of them reported eating fatty and fried foods twice a week or more frequently. Finally, 13.74% of participants reported drinking sweet beverages twice a week or more. Majority of participants stated that they had experience some sort of depression (41.22%) and anxiety (35.88%). The highest stress level (within the 0–3 score range) was related to their health with an average of 1.60, whereas the blood pressure, cholesterol, and blood sugar values above 140/90, 240 mg/dl, and 126 mg/dl respectively were observed in 25.95%, 16.03%, and 35.88% of participants, respectively. Moreover, 48.9% and 34.4% of the participants were overweight and obese, respectively (Table 1).

**Table 1:** Risk factors of cardiovascular disease in participants based on the centers for disease control and prevention framework for health risk assessments (n = 131)

Dimensions (n = 15)	Questions		Mean (SD)	n (%)
Physical inactivity	Mean days of physical activity in a week (per days)		0.82 (1.8)	
	Mean time of ‏activity (per min)		7.59 (16.37)	
	Severity (0–4)	Not exercising		103 (78.6)
		Light exercise		13 (9.9)
		Moderate exercise		12 (9.2)
		Heavy exercise		2 (1.)
Smoking	Smoking in past 30 days	Yes		34 (26)
		No		97 (74)
Alcohol	Drinking alcohol in past 7 days	Yes		7 (5.3)
		No		124 (94.7)
	If yes; how many times a week?		0.67 (0.27)	
	Driving after drinking?	Yes		4 (3.1)
		No		127 (96.9)
Nutrition	Usage of vegetables and fruits	Don’t use		6 (4.58)
		Once a week		36 (27.48)
		Twice and more		89 (67.94)
	Usage of high fiber or whole grain foods	Don’t use		0
		Once a week		40 (30.5)
		Twice and more		91 (69.5)
	Usage of fatty foods	Don’t use		46 (35.11)
		Once a week		53 (40.46)
		Twice and more		32 (24.43)
	Usage of sugar-sweetened (not diet)	Don’t use		73 (55.73)
		Once a week		40 (30.53)
		Twice and more		18 (13.74)
Seat belt	Fasten your seat belt	Yes		92 (70.2)
		No		39 (29.8)
Depression	In the past 2 weeks, how often have you felt down, depressed, or hopeless? ‏	Almost all of the time		23 (17.56)
		Most of the time		25 (19.08)
		Some of the time		54 (41.22)
		Almost never		29 (22.14)
	In the past 2 weeks, how often have you felt little interest or pleasure in doing things?	Almost all of the time		11 (8.4)
		Most of the time		30 (22.9)
		Some of the time		68 (51.91)
		Almost never		22 (16.79)
Anxiety	In the past 2 weeks, how often have you felt nervous, anxious, or on edge? ‏	Almost all of the time		18 (13.74)
		Most of the time		37 (28.24)
		Some of the time		47 (35.88)
		Almost never		29 (22.14)
	In the past 2 weeks, how often were you not able to stop or control your worrying?	Almost all of the time		20 (15.27)
		Most of the time		29 (22.14)
		Some of the time		58 (44.27)
		Almost never		24 (18.32)
Stress about (0–3)	Your health		1.6 (1.07)	
	Finances issues		1.44 (1.09)	
	Family or social relationships		1.48 (1.04)	
	Work		0.97 (1.13)	
Social support	Mean of social and emotional support		3.26 (0.86)	
General health (1–5)	Your idea about your health		2.71 (1.04)	
	Your idea about the condition of your mouth and teeth		2.25 (0.94)	
Sleep	Mean time of sleep		6.04 (1.85)	
Pain	In the past 7 days, how much pain have you felt?	None		3 (2.3)
		Some		95 (72.5)
		A lot		33 (25.2)
Pain severity (0–10)			6.47 (1.93)	
Self-care	In the past 7 days, did you need help from others to perform everyday self-care activities?	Yes		8 (6.1)
		No		123 (93.9)
ADLs	In the past 7 days, did you need help from others to take care of activity of daily living?	Yes		22 (16.8)
		No		109 (83.2)
Blood pressure	If your blood pressure was checked within the past year, What was it when it was last checked?	Low or normal (≤ 120/80)		58 (44.27)
		Borderline (120/80 to 139/89)		39 (29.77)
		High (≥ 140/90)		34 (25.95)
*Biometrics measures*				
Cholesterol	If your cholesterol was checked within the past year, what was your total cholesterol when it was last checked?	Desirable (below 200)		61 (46.56)
		Borderline high (200–239)		38 (29.01)
		High (240 or higher)		21 (16.03)
		Don’t know/not sure		11 (8.40)
Blood glucose	If your glucose was checked, what was your fasting blood glucose level the last time it was checked?	Desirable (below 100)		56 (42.75)
		Borderline high (100–125)		23 (17.56)
		High (126 or higher)		47 (35.88)
		Don’t know/not sure		5 (3.82)
Overweight/Obesity	BMI	< 20		3 (2.3)
		20–25		19 (14.5)
		25.1–30		64 (48.9)
		> 30		45 (34.4)

ADLs, Activities of daily livings; BMI, body mass index

### Analysis of illness perception

The mean of the illness perception was reported to be 43.49% (9.56) within the 0–80 range. Table 2 demonstrates the mean of each expression. The highest mean was related to the dimension of treatment control (6.83), whereas the lowest mean came from the understanding dimension (3.77). In addition, a good number of participants stated that the causes of illness were stress (36; 27.5%) and family issues (30; 22.9%).

**Table 2:** Mean, standard deviation and range for the 8 items of the brief illness perception questionnaire (B-IPQ) (n = 131)

Dimension (Item)	Mean (SD)	Range
Consequences (How much does your illness affect your life?)	6.64 (2.24)	2–10
Timeline (How long do you think your illness will continue?)	6.19 (2.64)	0–10
Personal control (How much control do you feel you have over your illness?)	4.58 (2.15)	0–10
Treatment control (How much do you think your treatment can help your illness?)	6.83 (2.21)	2–10
Identity (How much do you experience symptoms from your illness?)	6.28 (2.05)	0–10
Concern (How concerned are you about your illness?)	6.67 (2.29)	0–10
Understanding (How well do you feel you understand your illness?)	3.77 (2.22)	0–9
Emotional response (How much does your illness affect you emotionally	6.09 (2.36)	0–10
Total mean score	6.21 (1.36)	2.43–9.43
Total sum score	43.49 (9.56)	17–66

### Relationship between risk factors and illness perception

Table 3 shows the correlations between IHD risk factors (continuous variables) and the illness perception. Accordingly, the number of days of physical activities (r =  − 0.21, *p* = 0.014) and the duration of sports activities (r =  − 0.28, *p* = 0.001) had inverse significant correlations with illness perception. This meant that the less often participants engaged in physical activities, the higher the illness perception score; thus, they regarded the illness more threatening. Furthermore, the use of fatty foods (r =  − 0.17, *p* < 0.048) and sugar sweetened drinks (r =  − 0.22, *p* < 0.011) had inverse significant correlations with illness perception. In other words, when patients perceive the illness as more threatening, the total score of illness perception increases and the use of fatty foods and sugary drinks will decrease. There was also an inverse significant correlation between sleep and the total score of illness perception (r =  − 0.24, *p* = 0.005). In other words, the less sleep the patients reported, the higher the total score of illness perception; thus, the illness became more threatening. Also, the total score of the illness perception had direct significant correlations with cholesterol (r = 0.23, *p* = 0.006), glucose (r = 0.19, *p* = 0.025), systolic blood pressure (r = 0.37, *p* < 0.001), and diastolic blood pressure (r = 0.26, *p* = 0.002). In other words, the total score of illness perception increased as these biometric values increased, with patients perceiving the illness as more threatening. The findings reported no significant relationships between the total score of illness perception and other IHD factors or BMI (*p* > 0.05) (Table 3). Table 4 depicts the correlations between IHD risks (categorical variables) and illness perception. Participants’ total illness perception score had significant correlations with depression (*p* < 0.001), anxiety (*p* = 0.001), health-related stress (*p* < 0.001), financial issues (*p* < 0.001), family or social relationships (*p* = 0.003), and general health (*p* < 0.001). The subgroups of each of these variables were compared in terms of the total scores of illness perception by the LSD post hoc test. Results showed the total score of illness perception to be significantly higher in patients who felt more depressed, anxious, and stressed about their health, financial problems and family or social relations. Therefore, these patients were seen to perceive the illness as more threatening. At the same time, patients who perceived their general health as weaker had significantly higher scores of the illness perception. In other words, these patients also considered the illness more threatening. Other variables such as age, sex, and level of education did not influence on the illness perception.

**Table 3:** Correlations between illness perception and Health Risk assessment (continuous variables)

Dimensions of health risk assessments	Illness perception/Subscales r (p-value)								
	Consequences	Timeline	Personal control	Treatment control	Identity	Concern	Coherence	Emotion	Total mean score
Mean days of physical activity in a week	− .05 (.543)	− .25 (.003)	− .14 (.112)	− .16 (.065)	.02 (.813)	− .15 (.071)	− .16 (.067)	− .11 (.203)	− .21 (.014)
Mean time of physical activity in a day	− .04 (.629)	− .32 (< 0.001)	− .28 (.001)	− .19 (.029)	.02 (.763)	− .17 (.054)	− .27 (.002)	− .11 (.190)	− .28 (.001)
Drinking alcohol in past 7 days	.02 (.788)	− .12 (.147)	− .20 (.018)	− .18 (.035)	.02 (.795)	− .05 (.523)	− .09 (.272)	− .12 (.159)	− .15 (.080)
Usage of fruits and vegetables in past 7 days	.02 (.792)	− .08 (.314)	− .18 (.031)	− .10 (.253)	.12 (.151)	− .04 (.645)	− .24 (.005)	− .01 (883)	− .10 (.228)
Usage of high fiber or whole grain foods in past 7 days	− .01 (.888)	− .03 (.690)	− .03 (.700)	− .11 (.204)	.10 (.224)	.07 (.392)	− .15 (.080)	.10 (.236)	− .01 (.895)
Usage of fatty foods in past 7 days	− .15 (.076)	− .04 (.605)	− .25 (.003)	− .15 (.083)	− .10 (.227)	− .04 (.639)	− .15 (.082)	.02 (.740)	− .17 (.048)
Usage of sugar-sweetened (not diet) in past 7 days	− .05 (.540)	− .09 (.300)	− .31 (< .001)	− .28 (.001)	− .06 (.451)	− .08 (.323)	− .24 (.004)	.02 (.785)	− .22 (.011)
Severity of pain	.04 (.612)	− .10 (.245)	.05 (.537)	− .05 (.536)	.48 (< 0.001)	.14 (.104)	− .03 (.700)	.15 (.088)	.12 (.144)
Mean time of sleep	− .09 (.278)	− .16 (.068)	− .27 (.001)	− .13 (.131)	− .06 (.487)	− .14 (.110)	− .10 (.245)	− .21 (.012)	− .24 (.005)
Mean degree of cholesterol	.19 (.029)	.13 (.137)	− .01 (.886)	.12 (.160)	.23 (.008)	.27 (.002)	− .02 (.743)	.24 (.004)	.23 (.006)
Mean degree of glucose	.13 (.125)	.23 (.008)	.00 (.924)	.20 (.020)	.08 (.323)	− .00 (.994)	.21 (.012)	.06 (.491)	.19 (.025)
BMI	.13 (.126)	.16 (.067)	.01 (.854)	.16 (.057)	.00 (.952)	.14 (.104)	.02 (.770)	.15 (.080)	.17 (.051)
Systolic blood pressure	.31 (< 0.001)	.40 (.000)	.10 (.222)	.21 (.016)	.28 (.001)	.22 (.011)	− .11 (.200)	.32 (< 0.001)	.37 (< 0.001)
Diastolic blood pressure	.30 (< 0.001)	.27 (.002)	.02 (.786)	.10 (.249)	.23 (.008)	.15 (.072)	− .18 (.037)	.339 (< 0.001)	.26 (.002)

**Table 4:** Correlations between illness perception and health risk assessment (categorical variables)

Dimensions of health risk assessments	Cardiovascular Health Behaviors (*p*-value)								
	Consequences	Timeline	Personal control	Treatment control	Identity	Concern	Coherence	Emotion	Total mean score
Smoking (during past month)	.986	.635	.008	.055	.329	.955	.031	.497	.308
Depression	.034	.002	.049	.003	.024	.001	.699	< .001	< .001
Anxiety	.015	.004	.492	.029	.002	< .001	.093	< .001	.001
Stress related to health	< 0.001	.253	.382	.048	< .001	< .001	.113	< .001	< .001
Stress related to financial objects	.007	.046	.371	.031	.017	< .001	.316	.001	< .001
Stress related to family or social relationships	.002	.321	.792	.189	.003	< .001	.064	< .001	.003
Stress related to your job	.021	.993	.002	.883	.533	.084	.005	.023	.605
General health	.014	.006	.004	.072	.007	< .001	.015	< .001	< .001

## Discussion

This study aimed to determine heart disease risk factors and their correlations with illness perception among patients with an initial diagnosis of AMI who were undergoing PCI. The mean age of patients was low, with male and female patients averaging 53.25 and 57.28 years old, respectively. This finding is consistent with findings reported in a meta-analysis by Poorzand et al. [31]*.* The history of smoking over the past 30 days and history of familial heart diseases were positive in more than one-fourth and more than half of the participants, respectively. The familial history of positive CVDs is known to increase the chance of early emergence of heart disease by 2.35 [31]. In study of Gharios and colleagues [32], also a parental history of CHD, especially before 60 years, best predicted cardiovascular mortality. Findings of this study which include a positive history of co-morbid conditions such as HTN, DM, and HPL in most of the participants with a history of familial heart diseases, some of whom had two or three of those conditions simultaneously, lend credence to the fact that, CVD is a major cause of mortality in diabetic patients and that many other factors (*e.g.*, hypertension) play a key role in the high prevalence of CVDs [33]. Blood pressure increases in diabetic patients are known to be twice more than in non-diabetic patients and patients with hypertension are often resistant to insulin and are more prone to the risk of diabetes than normal people [34]. A high percentage of CVDs and CVD-caused deaths is attributed to the adjustable risk factors, the most important of which are metabolic factors [14]. The findings from this study that most participants engaged in no physical activities, nearly half of them were overweight and more than one-third of them were obese, point to the fact that they are prone to metabolic syndrome, the latter been seen as an important factor seen in several similar studies from Iran and all over the globe [35, 36]. Mirzaei et al*.* conducted a study in Yazd, Iran and reported that lack of physical activities and unhealthy eating habits were among the most prominent risk factors of CVDs in a healthy society [37]. They also reported that 26.5% of Iran’s healthy population would be prone to the high risk of CVDs based on the Framingham risk score (FRS) [38]. The onset age of CVDs is ten years earlier in Iran than in the developed countries [39]. The growing CVD epidemic of the recent 40 years in Iran might be correlated with socioeconomic developments, changes in diets, insufficient physical activities, industrialization and urbanization, increased life expectancy, increased metabolic and physical risk factors, insufficient and less cost-effective access to early care and treatment, and low adaptability for economic reasons and psychological problems [40]. According to the findings of this meta-analysis, the most prevalent and strongest risk factors of premature coronary artery disease (PCAD) in the Iranian population are type 2 DM, dyslipidemia, familial history of CAD, smoking, hypertension and higher values of BMI were reported for patients diagnosed with PCAD [31]. These findings indicate the important roles that high BMI and a positive familial history of CAD play in the development of atherosclerotic CAD, especially with respect to the mean age of about 55 years as seen in more than half of study participants who experienced AMI for the first time. The results of one systematic review reveal that diabetes mellitus type 2, familial history of CAD, dyslipidemia, smoking, and hypertension are significantly and positively correlated with CAD in young adults compared with the healthy population of Iran [34] while in other studies, the prevalence of IHD risk factors was subject to change depending on the people’s lifestyle habits, income and health systems of countries [41, 42]. A similar study in Greece reported the prevalence of hypertension, hyperlipidemia, obesity, and severe diabetes [43] while another study in Poland reported that a history of current or previous habit of smoking was associated with a high prevalence rate of CVD and CV risk factors [44]. Moo-Sik et al*.* evaluated the scores of CVD risk factors in patients under PCI with no history of CVDs through the FRS and concluded that some of the conventional risk factors (*e.g.*, high BMI) increased within a 17-year period. They also demonstrates that although traditional FRS and its associated predicted 10-year cardiovascular risk declined over time, the prevalence of risk factors increased in patients undergoing PCI. The study suggests the need for a new risk-factor assessment in this patient population [45]. Apparently, the *HRA* framework can be employed to evaluate the IHD risks and provide caregivers with more information regarding risks and corrective measures.

The total mean score of illness perception was high among participants of this study, something which indicates that they considered the illness a threat. Other findings of this study included the low score of illness perception in the dimensions of personal control and understanding. By contrast, the participants had high illness perception scores in the dimensions of concern and treatment control. Lotfi et al*.* reported a similarly high total mean score of illness perception [20] as did another study from Indonesia which reported a high total mean score of illness perception and the score of understanding and personal control to be average and low, respectively, which is consistent with findings from this present study [46]. In a study by Petriček et al*.*, the dimension of understanding had the lowest mean score and was a significant predictor of BMI [47]. In related studies, the threatening perception of the illness was associated with the numerous outcomes of patients such as a reduced quality of life, lower levels of health and higher levels of emotional distress [48], less frequent presence in cardiac rehabilitation programs [49], low adherence to prescribed medications, sports, and recommended diets [50], and high mortality [51]. Thus, training and counselling programs are required to modify and improve the illness perception of participants in this current study. Ashour et al*.* reported that the low perception of personal control and the high perception of disease symptoms could predict the highly perceived learning needs in patients after a PCI [52].

With the findings of this study, it is seen that the total mean score of the illness perception had inverse correlations with physical activity, use of fatty and fried foods, sugar sweetened drinks, sleep, and general health. In other words, patients who perceived the disease as more threatening reported fewer physical activities as well as less use of fatty and fried foods and sugary drinks. Consistent with the findings of this present study is another study which reported that a more threatening view of an illness was correlated with the better diet management [46]. However, findings of some previous studies indicate contradictory results regarding this relationship between illness perception and healthy behavior. For instance, Mosleh et al*.* predicted better illness perception, better adherence to physical activities, and better adherence to treatment [50]. Nonetheless, Gauro et al*.* in Nepal reported an inverse correlation between illness perception and cardiovascular health behavior [21], which show that opinions about an illness require psychological training intervention to improve disease management methods and enhance adherence to healthy behaviors [50]. Patients with more threatening views of their diseases reported fewer sleep hours, higher levels of anxiety, stress, and depression, and lower levels of general health. Yeom and Shin reported that the illness perception could be correlated with sleep hours and disease-caused stress and that stress could have a mediating role in the correlation between the illness perception and sleep [53]. In other words, the negative effect of illness perception on sleep can be mitigated by regulating stress. It is also necessary to develop intervention programs that can reduce the stress related with illness perception among patients. Doi-Kanno and H. Fuahori reported that negative opinions about illness and other factors such as the physical activity patterns, use of fat, smoking, stress, and anxiety after the PCI could predict depression in patients after an MI [54]. According to the findings of this study, the total score of the illness perception had direct correlation with biometric values such as cholesterol, blood glucose, and blood pressure. In other words, increasing each of these biometric values would make the illness more threatening. Brewer et al*.* found a correlation between the better identification of an illness and the better control of cholesterol as well as adherence to drugs [55]. Other studies have reported that illness perception was correlated with the self-care behaviors aimed at improving health and moderating lifestyle [56, 57]. In a study by Goldman, participants believed that use of anti-hypertensive drugs would be preferable to cholesterol-lowering drugs and considered cholesterol levels less important than hypertension [58]. Therefore, in the light of these study findings, it is necessary to develop national training and health checkup programs aimed at teaching patients, providing them with lifestyle modification strategies [59], and improving the patient–clinician relationship [60] by enhancing patients’ knowledge about the role of biometric values and other risk factors in the development of CVD.

## Conclusion

In this study, participants experienced most of the IHD risk factors and had high mean scores of concern about the illness and its outcomes in terms of the illness perception. This might lead to psychological outcomes such as stress, anxiety, and depression. The correlations of the illness perception with some lifestyle components such as physical activity and diet as well as with biometric values indicate the necessity of paying more attention to training patients and providing them with consultation to improve the perception of CVDs as a manageable illness. As far as it is known, this is the first study to employ the CVD risk assessment framework published by the CDC, USA in Iran. The positive familial history of CVDs, diabetes, and hypertension depicts the necessity to paying more attention to these patients in terms of screening for hypertension, diabetes, and dyslipidemia. Health policies in Iran and similar climes should be focused on the risk factors having major roles in preventing CVDs and mortality with further emphasis on the most important factors in specific groups such as patients with positive familial history, diabetes, and hypertension. Doctors, nurses and other clinicians should be trained to execute the crucial role of ensuring that patients are educated properly on managing lifestyles in a bid to mitigate the psychological outcomes resulting from myocardial infraction and its treatments. Evidence from this study about the relationship between illness perception and some psychological, and modifiable lifestyle factors can provide ideas for other studies. In other words, our results suggest that the design of an educational intervention study and its consequences in the disease follow-up phase be done.

One of the limitations of this study is the cross-sectional nature of it, the relationships shown between HRA questionnaire’s dimensions with illness perception cannot accurately reflect the causal relationship. Another limitation of the study was convenience of the sampling that reduces the generalizability of the results. In addition, in current study, patients who experienced first myocardial infractions, received PCIs for the first time could recruit in the study. Therefore, as a limitation, this study may not be the representative of patients with AMI in Iran in general.

## Data Availability

The data that support the findings of this study are available from the authors upon reasonable request.

## Abbreviations

ADL: Activities of daily living
AMI: Acute myocardial infarction
ANOVA: Analysis of variance
BMI: Body mass index
BP: Blood pressure
CCU: Cardiac care unit
CDC: Centers for disease control and prevention
CHD: Coronary heart disease
CVD: Cardiovascular disease
DBP: Diastolic blood pressure
DM: Diabetes mellitus
HPL: Hyperlipidemia
HRA: Health risk assessments
HTN: Hypertension
IHD: Ischaemic heart diseases
IPQ: Illness perception questionnaire
IPQ-B: Brief illness perception questionnaire
LSD: Least significant difference
MI: Myocardial infraction
PCI: Percutaneous coronary intervention
SBP: Systolic blood pressure
